# Effects of service changes affecting distance/time to access urgent and emergency care facilities on patient outcomes: a systematic review

**DOI:** 10.1186/s12916-020-01580-3

**Published:** 2020-05-20

**Authors:** Duncan Chambers, Anna Cantrell, Susan Baxter, Janette Turner, Andrew Booth

**Affiliations:** School of Health and Related Research (ScHARR), University of Sheffield, Sheffield, UK

**Keywords:** Ambulance services, Distance to care, Emergency care, Emergency departments, Service reconfiguration, Systematic review, Urgent care

## Abstract

**Background:**

Reconfiguration of urgent and emergency care services often increases travel time/distance for patients to reach an appropriate facility. Evidence of the effects of reconfiguration is important for local communities and commissioners and providers of health services.

**Methods:**

We performed a systematic review of the evidence regarding effects of service reconfigurations that increase the time/distance for some patients to reach an urgent and emergency care (UEC) facility. We searched seven bibliographic databases from 2000 to February 2019 and used citation tracking and reference lists to identify additional studies. We included studies of any design that compared outcomes for people with conditions requiring emergency treatment before and after service reconfiguration with an associated change in travel time/distance to access UEC. Studies had to be conducted in the UK or other developed countries. Data extraction and quality assessment (using the Joanna Briggs Institute checklist for quasi-experimental studies) were undertaken by a single reviewer with a sample checked for accuracy and consistency. We performed a narrative synthesis of the included studies. Overall strength of evidence was assessed using a previously published method that considers volume, quality and consistency.

**Results:**

We included 12 studies, of which six were conducted in the USA, two in the UK and four in other European countries. The studies used a variety of observational designs, with before–after and cohort designs being most common. Only two studies included an independent control site/sites where no reconfiguration had taken place. The reconfigurations evaluated in these studies reported relatively small effects on average travel times/distance.

**Discussion:**

For studies of general UEC populations, there was no convincing evidence as to whether reconfiguration affected mortality risk. However, evidence of increased risk was identified from studies of patients with acute myocardial infarction, particularly 1 to 4 years after reconfiguration. Evidence for other conditions was inconsistent or very limited.

**Conclusions:**

We found insufficient evidence to determine whether increased distance to UEC increases mortality risk for the general population of people requiring UEC, although this conclusion may not extend to people with specific conditions.

## Background

The impact of large-scale changes to the delivery of health services (often referred to as service reconfiguration) is important to health professionals, health service managers, and patients and the public. Programmes of service reconfiguration in the English National Health Service (NHS) are currently being implemented at a local level to deliver new models of care such as integrated care systems (ICS) [1]. Proposed reconfigurations may increase travel time and/or distance for some patients to reach their nearest hospital emergency department (ED) or other urgent and emergency care (UEC) facility, for example by closing EDs or replacing a full ED with an urgent care centre or minor injury unit. The rationale for reconfiguration is that by concentrating resources in fewer specialist centres, patients with severe acute conditions will receive better quality care and achieve better outcomes. Patients with less serious conditions will be catered for by a local urgent care centre/minor injury unit or by triage at a large ED.

Many communities value their local UEC services and perceive that proposed changes which may increase travel time and/or distance could worsen outcomes for patients, particularly those requiring emergency medical or obstetric care [2]. In addition to increased morbidity/mortality, potential harmful effects of reconfiguration could include financial costs for patients/families; overcrowding and longer waiting times at large EDs; environmental effects of extra road journeys; and disruption to existing clinical relationships and pathways. Commissioners and service providers need evidence regarding the impacts of reconfiguration not only on patient outcomes, but also for the wider healthcare system [3]. For example, commissioners may have questions about effects on other provision such as ambulance and community-based services. Providers may face difficulties in staffing other services if they are no longer providing emergency care.

The recent closED study [3] analysed data from five locations in England where emergency departments (EDs) were downgraded between 2009 and 2011. While the authors found no evidence of an impact on mortality (despite patients having to travel further to access an emergency facility), the study did detect evidence of an effect on the UEC system as a whole, such as an increased burden on emergency care providers. The aim of this systematic review was to assess the international evidence on the effects of reconfiguration that increases the distance people have to travel (and/or the time taken) to access emergency care. We defined reconfiguration to include large-scale system change, such as relocation of hospitals, (re) location of specialist care, and changes in provision of urgent/emergency/out-of-hours care [2]. This definition would exclude small-scale change, for example at hospital ward level or within a general practitioner (GP) practice.

The work formed one strand of a larger project, funded by the UK National Institute for Health Research, and the full technical report will be published in due course (Chambers et al., *Health Services and Delivery Research*, in preparation).

## Methods

The protocol for this review was registered prospectively on the PROSPERO database (registration number CRD42019123061). The research question was: what is the evidence regarding effects on patients and the health system of service reconfigurations that increase the time/distance for some patients to reach an UEC facility? A list of potentially time-sensitive conditions requiring treatment at a UEC facility was developed in advance (see inclusion and exclusion criteria below). The list prioritised conditions more likely to be affected by service reconfiguration or requiring a decision as to whether to travel further to reach a more specialist facility. However, this list was not intended to be exhaustive and studies of other conditions were included if they met the other inclusion criteria.

### Literature search and screening

A comprehensive literature search was conducted in February 2019. The search was developed on MEDLINE and utilised MesH and free-text terms. The search comprised four broad facets—Emergency Care, Rural or Island services, Service reconfiguration and potentially relevant emergency conditions. Search terms covering access, distance, and travel time were also included. The search was limited to papers from 2000 to February 2019 and in English. The MEDLINE search is provided in Additional file 1 with details of how the different facets of the search strategy were combined.

The MEDLINE search was translated to the other databases. The databases searched were MEDLINE via OvidSP; Embase via Ovid; Cochrane Database of Systematic Reviews via Wiley Interscience; Cochrane Central Register of Controlled Trials via Wiley Interscience; CINAHL (Cumulative Index to Nursing and Allied Health Literature) via EBSCO; HMIC (Health Management Information Consortium) via OpenAthens; and Web of Science (Science Citation Index and Social Sciences Citation Index) via Web of Knowledge via ISI.

Citation tracking of included studies was performed on Web of Science (WOS) and Google Scholar in April 2019. Given the diffuse nature of the topic and associated terminology, the reference lists of all included articles were manually screened to identify additional studies.

Search results were imported into EndNote X8.2 (Philadelphia, USA: Clarivate Analytics), and automatic and manual deduplication was conducted. Records were imported into EPPI-Reviewer 4 software (London, UK: EPPI Centre) for screening, data extraction, and quality assessment. The search results were screened against the inclusion criteria by a single reviewer, with a 10% sample screened by a second reviewer. Uncertainties were resolved by discussion amongst the review team.

### Inclusion and exclusion criteria

#### Population

Population includes adults or children with conditions that require emergency treatment including but not limited to acute myocardial infarction (AMI), stroke, major trauma, severe exacerbations of asthma, chronic obstructive pulmonary disease or complications during pregnancy and the neonatal period. In practice, eligible studies could include data on any patient wishing to access an UEC facility.

#### Intervention

Intervention includes changes to the delivery of healthcare services (service reconfiguration) which have an effect on the time or distance for patients to access an UEC facility. The review included reconfigurations that have an effect on access to any urgent and emergency care services including ambulance services, maternity services and hospital emergency departments.

#### Comparison

Comparison entails outcomes (from studies with or without control sites) before and after a service reconfiguration which has an effect on time/distance to UEC.

#### Outcomes

Outcomes entail any quantitative or qualitative outcomes for patients including mortality/morbidity, or other perceived or measured effects. Also outcomes or impacts on the health system such as non-transportation to hospital, emergency admissions, increase or decrease in contacts/service usage.

#### Setting

Setting includes the UK and other developed countries. Absolute travel distances and density of population (which will affect distribution and density of healthcare facilities) were taken into account in assessing applicability of findings to the UK.

#### Study design

Studies of any design were eligible for inclusion.

##### Other inclusion criteria

- Literature published since 2000
- Literature published in English
- Grey literature in the form of service evaluations or reports from the UK

##### Other exclusion criteria

- Studies that describe reconfigurations or initiatives without providing any quantitative or qualitative data
- Conceptual papers and projections of possible future developments
- Studies conducted in low- or middle-income country health systems
- Studies conducted in high-income countries that are not considered comparable to the UK health system
- Studies of air ambulance services were excluded because these services are not funded by the NHS in England
- Theses, conference abstracts, articles in professional magazines, books and book chapters

### Data extraction and quality assessment

We extracted and tabulated key data from the included studies, including study design, population/setting, results and author-reported key limitations. The full data extraction template is provided in Additional file 2. Data extraction was performed by a single reviewer with a 10% sample checked by a second reviewer for accuracy and consistency.

Quality (risk of bias) assessment was undertaken using the Joanna Briggs Institute checklist for quasi-experimental studies [4]. This nine-question checklist was chosen because the meaning of included items was considered easily understandable and because the questions are applicable to a wide range of non-randomised study designs. Quality assessment was performed by a single reviewer with a 10% sample checked for accuracy and consistency.

### Evidence synthesis

We performed a narrative synthesis structured around the pre-specified research questions and outcomes. We first described the characteristics of the group of studies as a whole. We then summarised the results in terms of the types of conditions included (e.g. general UEC population, acute MI, trauma). Further analyses assessed the relevance of the study setting to the UK health system and explored rural, compared to urban and suburban, settings. The narrative synthesis was drafted by the first author and revised with input from all the authors.

Summary table reports were generated from extracted data using the EPPI-Reviewer program. Overall strength of evidence was assessed using a previously described method [5]. Evidence was rated as comparatively ‘stronger’, ‘weaker’, ‘inconsistent’ or ‘very limited’ based on volume, strength and consistency. Specifically, ‘stronger evidence’ represented generally consistent findings in multiple studies with a comparator group design or comparative diagnostic accuracy studies; ‘weaker evidence’ represented generally consistent findings in one study with a comparator group design and several non-comparator studies or multiple non-comparator studies; ‘very limited evidence’ represented an outcome reported by a single study; and finally, ‘inconsistent evidence’ represented an outcome where fewer than 75% of studies agreed on the direction of effect. All studies included in the review were included in the analysis of overall strength of evidence.

### Public and patient involvement

We elicited input from the Sheffield Evidence Synthesis Centre public advisory group, who contributed across all the stages of the review including helping to understand the importance of the question to patients and the public and interpreting the findings. The advisory group emphasised how international health systems may not be directly comparable to the UK and encouraged the research team to be clear regarding applicability of international evidence.

## Results

### Study selection

**Fig. 1:**
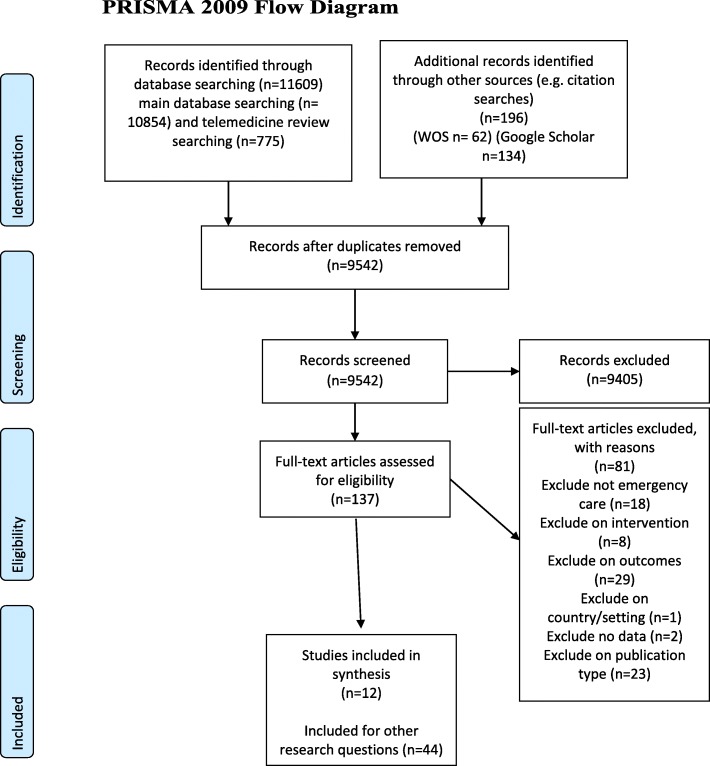
PRISMA flow diagram

The PRISMA flow diagram (Fig. 1) summarises the study selection process. Calculation of the Kappa coefficient demonstrated good agreement between reviewers for the sample of double screened records (*K* = 0.729, 95% CI, 0.542–0.916). Reasons for studies being excluded at the full-text stage included their covering access to services generally, not specifically emergency care; the intervention was not relevant (e.g. public access defibrillators); or the study discussed changes to services without relating outcomes to travel time or distance.

### Characteristics of included studies

Table 1 summarises the characteristics of the included studies. Six studies were conducted in the USA, with only two [3, 13] from the UK. The remaining studies were conducted in other European countries; there were no studies from Canada, Australia or New Zealand.

**Table 1:** Characteristics of included studies

**Study ID**	**Country**	**Study design**	**Condition**	**Intervention**	**Sample source**	**Sample size**	**Length of study**
Avdic 2016 [6]	Other Europe Sweden	Controlled observational Analysis of linked administrative datasets	Acute MI	Hospital ED closure	Administrative registers obtained from the Swedish National Board of Health and Welfare (hospitalisations and deaths)	Approximately 374,000 events	21 years (1990–2010)
Combier 2013 [7]	France Burgundy region	Uncontrolled observational Before–after study	Obstetric/neonatal complications	Obstetric unit closure	Hospital discharge summary data for all deliveries from 22 weeks’ gestation in the region’s maternity units	111,001 deliveries	10 years (2000–2009)
El Sayed 2012 [8]	USA	Uncontrolled observational Before–after study	General emergency care	Hospital ED merger	Routinely collected EMS and ED data	5338 EMS transports; 21,685 ED visits	3 months (June 1 to August 262,010)
Hansen 2011 [9]	Other Europe Denmark	Uncontrolled observational Before–after study	General emergency care	Hospital ED closure	Danish National Person Registry including all Danish residents	21,000 residents of Viborg county (2300 from Morso)	7 years (1997–2003)
Hsia 2012 [10]	USA California	Controlled observational Cohort	General emergency care Acute MI, stroke, sepsis and asthma/COPD	Hospital ED closure	California Office of State-wide Health and Planning Development database, combined with information on ED closures by year between 1999 and 2009	785,385, of whom 67,577 (8.6%) experienced an increase in distance to ED care as a result of an ED closure	11 years (1999 to 2009)
Hsia 2014 [11]	USA	Other cross-sectional comparison of existing datasets, compared at T1 and T2 10 years later.	Major trauma Acute trauma aged 20 or older.	Trauma unit closure	Database of trauma centres open at T1 and 10 years later at T2. Patient discharge database. Household demographic database.	266,023 had no increased drive time, 5122 had increased drive time.	Compared 1999 to 2009
Knowles 2018 [3]	UK	Controlled observational Interrupted time series	General emergency care	Hospital ED closure or downgrade	ONS, HES, ambulance dispatch records	Unable to locate, refers to areas only	Two years pre closure and 2 years post closure.
Mustonen 2017 [12]	Other Europe Finland (Vantaa, Finland’s third-largest city, with approximately 182,000 inhabitants)	Controlled observational Controlled before–after study	General emergency care	Primary care ED closure	Electronic health records plus monthly mortality statistics by age groups	Unclear (34,000 inhabitants in area with ED closure)	4 years (February 2004 to December 2007)
Roberts 2014 [13]	UK England only	Uncontrolled observational National data on distance travelled to emergency care plus three case studies of local reconfiguration	General emergency care	Hospital ED closure or relocation	Hospital Episode Statistics plus data on ED attendances from every major (type 1) ED in England	13 million ED attendances and 5.4 million emergency admissions (2011/12)	10 years (2001/2 to 2011/12)
Shen 2012 [14]	USA	Controlled observational Difference in difference approach	Acute MI	Hospital ED closure or relocation	American hospital annual survey, database for California hospitals, Medicare claims	Unclear	4 years before change to 4 years after change to ED access
Shen 2016 [15]	USA	Controlled observational	Acute MI	Hospital ED closure or relocation	Medicare records, cost provider systems	1.35 million patients	90-day mortality reported in this paper
Yaghoubian 2008 [16]	USA California (Los Angeles County)	Uncontrolled observational Interrupted time series	Major trauma	Trauma centre closure	Patient records from prospectively collected database (Trauma and Emergency Medicine Information System)	14,996	9 years 2 months (January 1997 to 1 March 2006)

Six of the included studies focused on ED reconfiguration, providing data on patients with many different types of emergency conditions. Three looked specifically at patients with acute MI requiring access to percutaneous coronary interventions (PCI). Two studies examined the effects of service changes involving specialist trauma centres, and one looked at the effects of maternity unit closures in France.

The studies used a variety of observational designs, with before–after and cohort designs being most common. Knowles et al. [3] and Mustonen et al. [12] were the only studies that compared reconfiguration sites with independent control sites where no reconfiguration had taken place.

### Risk of bias

Results of the quality appraisal are presented in Additional file 3. Many of the studies were inherently at high risk of bias because of lack of an independent control group. The most common design was before–after and only four studies compared outcomes between settings with and without changes in distance/time [3, 6, 10, 12].

Most of the included studies were clear about the temporal relationship of the variables of interest (i.e. which was the ‘cause’ and which was the ‘effect’; Q1), although the issue was sometimes confused by the use of linked datasets. Similarity between populations being compared (Q2) varied across the studies. It was also sometimes unclear whether comparison groups were being treated similarly other than the intervention or exposure of interest (Q3). This related to differences over time as well as to studies recruiting clinically diverse populations. Absence of a separate independent control group (Q4) was noted in most of the studies and few studies carried out measurements at multiple time points before and after an intervention or exposure (interrupted time series design; Q5). Completeness of follow-up (Q6) did not show a clear pattern across studies. Most studies measured outcomes in a standard (Q7) and reliable (Q8) way, although again some exceptions were identified. Statistical analysis (Q9) was judged to be appropriate with the exception of one study which presented summary data without any statistical analysis [13]. As is the case for all observational studies, the possibility of unmeasured confounders affecting the results could not be ruled out.

### Effects on mortality

Most of the included studies reported changes in mortality rates following reconfiguration (Table 2). For the two large studies of general UEC populations, people experienced increases in time/distance of up to 33 miles [10] or 25 min [3]. However, most increases were considerably smaller (median less than 1 mile in Hsia et al. [10]) and neither study provided evidence of an effect on mortality. For patients with MI, increases of over 30 min were associated with significant increases in mortality, but in a large US study, only 0.2% of patients fell into this group [14]. Findings for trauma centre and maternity closures were less clear because of the small number/size of studies.

**Table 2:** Summary of key results on changes in travel distance/time and mortality following UEC service reconfiguration

**Study ID**	**Population**	**Comparison**	**Reported change in travel distance/time**	**Reported effect on mortality**
Hsia 2012 [10]	General UEC	Increased distance vs. no change	Median 0.8 miles (range 0.1 to 33.4)	OR 1.04 (95% CI 0.99 to 1.09)
Mustonen 2017 [12]	General UEC	ED closure vs. site with no closure	Not reported	No increase in any age group
Knowles 2018 [3]	General UEC	ED closure vs. control site with no closure; before vs. after closure within sites; sub-areas at each site with above vs. below median change in travel time	Increased travel time range by site: 0–19, 0–22, 0–14, 0–23 and 0–25 min	No statistically reliable evidence to suggest a change in the number of deaths following an ED closure in any site or on average across all sites
Avdic 2016 [6]	Acute MI	ED closure vs. sites with no closure	Median 13 km	Mean difference in survival to discharge 0.015 (72.4 vs. 74.9%)
Shen 2012 [14]	Acute MI	Increased travel time vs. no change	No change 89.2%; < 10 min 8.9%; 10–30 min 1.7%; > 30 min 0.2%	Increase in mortality rate for those with > 30 min increase: 1.72 percentage points at 7 days, 1.23 at 30 days, 2.58 at 90 days, 4.49 at 180 days and 5.65 at 1 year
Shen 2016 [15]	Acute MI	Increased travel time vs. no change	Categories as Shen 2012	Increase in mortality rate for those with > 30 min increase: 6.58 percentage points (95% CI 2.49 to 10.68) at 90 days and 6.52 (95% CI 1.69 to 11.35) at 1 year. Significant but less pronounced effect for 10–30 min increase, no effect for < 10 min
Hsia 2014 [11]	Trauma	Increased travel time vs. no increase (no change or decrease)	Average travel time to the nearest trauma centre was 47 min (interquartile range, 27–52) for patients who experienced an increase in travel time and 34 min (interquartile range, 23–35) for those who did not	OR for in-patient mortality 1.21 (95% CI 1.04 to 1.4) overall and 1.29 (95% CI 1.11 to 1.51) during the first 2 years after a closure
Yaghoubian 2008 [16]	Trauma	Before vs. after trauma centre closure	Median transport time 12 (interquartile range 8–17) vs. 13 (9–17) minutes	Mortality rate increased from 5.4 to 7.3% but was lower in the later period after adjusting for severity score (OR 0.69, 95% CI 0.49 to 0.97)
Combier 2013 [7]	Maternity	Before vs. after maternity unit closures	Mean time was estimated at 21 min in 2000 and at 24 min in 2009, while maximum time increased from 61 to 72 min	No significant association between travel time and stillbirth or perinatal mortality at any time point

In summary (Table 3), stronger evidence (derived from studies with control groups) did not support or refute the hypothesis that reconfiguration resulting in increased travel time/distance affected mortality rates. In other words, there was no evidence of an effect, making it difficult to draw firm conclusions from this evidence. By contrast, there was evidence of increased risk from studies restricted to patients with acute MI. Evidence for other conditions was inconsistent or very limited. It was notable that none of the included studies had collected data relating to stroke patients specifically (although people with stroke were an identifiable subgroup in the study by Hsia et al. [10]).

**Table 3:** Summary of evidence on mortality

**Population**	**Relevant studies**	**Evidence statement**	**Strength of evidence**	**Comments**
General UEC	Hsia 2012 [10] = Mustonen 2017 [12] = Knowles 2018 [3] =	No effect of reconfiguration on mortality	Stronger	Interpret as no evidence of an effect
Acute MI	Avdic 2016 [6] − Shen 2012 [14] − Shen 2016 [15] −	Increased mortality risk following reconfiguration	Stronger?	
Trauma	Hsia 2014 [11] − Yaghoubian 2008 [16] +	Unclear effect on mortality risk following reconfiguration	Inconsistent	
Maternity	Combier 2013 [7] =	Insufficient evidence	Very limited	

Controlled studies in bold; = means no significant difference in outcomes; + means better outcome with increasing distance; − means worse outcome with increasing distance; +/− varying results within study; ? results difficult to interpret in comparative terms

While the evidence on mortality for the trauma population was inconsistent overall, results from two studies suggested that trauma centre closure may impact negatively on outcomes at remaining trauma centres within a region [11, 16]. However, this finding may be of limited relevance to the UK, where the implementation of a network of trauma centres in recent years means that availability of trauma centres is matched to needs and significant reconfiguration resulting in closures is unlikely.

## Discussion

### Main findings

In practice, reconfiguration of UEC services in a publicly funded health system like the UK NHS sometimes means closure of EDs or downgrading by reducing the opening hours or the variety of services provided. This is generally considered likely to increase travel distance/time for the majority of patients in the affected area as well as the overall average distance/time to reach a suitable UEC facility. However, the studies included in this review suggested that such increases may be small (less than 1 mile or 10 min) for most people, with a small minority experiencing increases of 30 miles/30 min or more [3, 10, 14].

Overall, the studies found no convincing evidence as to whether increasing travel time or distance increased mortality risk for general populations of patients attending UEC facilities. The reconfigurations evaluated in these studies reported relatively small effects on average travel distance/time. This is representative of the situation in the UK (at least in England) where distances travelled to reach a UEC facility have remained fairly short and stable over time [13]. There was some evidence of an increased risk from studies restricted to patients with acute MI, while evidence for other conditions was inconsistent or very limited. This suggests the possibility that the effect of increased distance or time may be diluted in the general UEC population by the presence of patients with less serious conditions and minimal short-term risk of death. However, one of the largest studies found no change in in-patient mortality for either the population as a whole or subgroups with specific emergency conditions [12].

The implications for the health system as a whole of reconfiguring a key part of UEC might be conjectured as being substantial. For example, attendance at remaining EDs in the area may increase, EMS staff may be required to cover a larger catchment area, and hospitals may face difficulties in staffing other services if no longer providing emergency care means that they are perceived as less-prestigious places to work and provide reduced clinical and training opportunities.

It is important to note that the findings of this review suggest that the effects of service reconfiguration on outcomes (particularly patient outcomes) may be short-lived, with health systems adapting to the new situation in the subsequent few years. In the study by Avdic [8], effects of ED closures on acute MI mortality were only statistically significant for the first year after closure, and Shen et al. identified a 4-year transition period [14]. Efforts by healthcare commissioners and providers to mitigate the effects of reconfiguration may be key to minimising the effect of changes. Avdic referred to increased investment in both emergency service provision and prevention, although the study did not evaluate whether these actually occurred [6]. A study from the USA highlighted how early notice of an ED closure was followed by close working amongst providers to minimise the effect on the EMS system in the city [8]. Also in the USA, Yaghoubian et al. reported how changes in trauma centre staffing and organisation were put in place to prepare for the closure of a nearby centre [16]. The insights provided by these studies indicate the need for greater understanding of how health service stakeholders prepare for the system-wide impacts of changes that require patients to travel further for treatment [3].

Service reconfiguration is often advocated by decision-makers who argue that increased patient volume and/or specialisation in a smaller number of UEC facilities will increase the overall quality of patient care. This review did not directly address the relationship between volume of contacts and outcomes, as this area has been the subject of a large volume of research. However, one study included in our review attributed successful outcomes following a trauma centre closure partly to staff gaining experience from treating more patients [16]. When considering the influence of treatment by highly skilled specialist staff on patient outcomes, the substantial body of evidence for the benefits of transporting patients with stroke [17], AMI or severe trauma to specialist centres (which may be further away), rather than attending nearer non-specialist facilities, should be taken into account.

### Strengths and limitations

This systematic review was undertaken by an experienced team including both methodological and topic experts. We performed a thorough search for published literature published since 2000 including supplementary searching methods such as citation tracking. The review also benefitted from the input of an experienced public advisory group.

Because of resource constraints, we abbreviated the review process by using a single reviewer to perform study selection, quality assessment and data extraction, with checking of a 10% sample by a second member of the review team. Double independent performance of these stages was not a viable option but analysis of study selection revealed a high level of agreement amongst three reviewers. While there is a risk of some errors or subjective assessments, we do not believe these would have influenced the review’s main findings and conclusions.

Much of the research included in our review originates from non-UK settings, and we have tried to keep applicability in mind throughout. The US health system is organised and funded very differently from the UK NHS but there is no reason to suppose that this would affect the relationship between distance/time and outcomes for patients with a particular condition. Given the low quantity and quality of evidence we expected to include in the review, we made a pragmatic decision to include studies from the USA with appropriate caveats. Absolute distances and times of travel vary within countries, including the UK, but large countries such as the USA, Canada and Australia are likely to have longer travel times/distances on average outside urban areas. This is also true for some of the Scandinavian countries, where travel times can be long because the population is centred in fewer areas.

Interpretation of the findings of systematic reviews should be guided by the quality and strength of the included evidence. We have assessed methodological quality of the individual studies and overall strength of evidence for key findings using a scheme successfully employed in previous reviews. Some of the included studies were judged to be at relatively high risk of bias because of their observational design and the absence of an independent control group. On the positive side, most studies acknowledged and attempted to adjust for the influence of confounding factors and some were large and/or long term. In view of this uncertainty, we have been conservative in assessing the overall strength of evidence for effects and associations (see Table 3).

### Implications for further research

There is a need for further time series analyses along the lines of the closED study [3] to examine the longer-term effects of service reconfigurations on the whole UEC system and to take into account the impact of other service and technological changes over time. While such studies should ideally be controlled, uncontrolled time series also have some value and offer fewer logistical challenges.

Research is needed to better understand how local and regional health systems plan for, and adapt to, increases in travel distance/time. As suggested by other researchers [3], this could take the form of qualitative research and/or documentary analysis. The current programme of service reconfiguration provides opportunities for prospective studies across diverse settings. Research should aim to capture the perspectives of different stakeholders including health professionals, managers in both commissioner and provider organisations and the public.

Analysis of routine data will enable researchers to examine whether UEC reconfigurations reduce overall demand for ED care or merely displace demand to other parts of the health system. Data can also be used to examine the nature and extent of variation between different localities with a view to reducing unnecessary variation and improving overall quality of care.

Research is needed to assess patient outcomes other than mortality and hospital admission/length of stay. This could include effects of service reconfiguration on families who may incur additional social and financial costs because of increased travel distance/time to visit patients.

## Conclusions

This systematic review found no convincing evidence to support or refute the perception that service changes that increase average travel time or distance increase mortality risk for general populations of patients attending UEC facilities. Large observational studies suggested that increases are small for most of the population affected. There was some evidence of an increased risk from studies restricted to patients with acute MI, while evidence for other conditions was inconsistent or very limited.

The relatively low quality of much of the research suggests that findings should be interpreted cautiously. In particular, ‘no evidence of increased risk’ does not necessarily mean ‘evidence of no increase in risk’ as the finding could be overturned by further research in the future.

Research priorities include work to examine the longer-term effects of service reconfigurations on the whole UEC system and to better understand how local and regional health systems plan for and adapt to increases in travel distance/time.

At the time of completing this paper, health services worldwide were confronted with unprecedented pressure on UEC services as a result of the coronavirus (COVID-19) pandemic. The effects of this event on attitudes to UEC service provision and reconfiguration remain to be seen.

## Supplementary information

Additional file 1. Medline search strategy.

Additional file 2. Data extraction template.

Additional file 3. Quality assessment results.

## Abbreviations

AMI: Acute myocardial infarction
CINAHL: Cumulative Index to Nursing and Allied Health Literature
ED: Emergency department
EMS: Emergency medical services
HES: Hospital Episode Statistics
HMIC: Health Management Information Consortium
ICS: Integrated care system
NHS: National Health Service
ONS: Office for National Statistics
PRISMA: Preferred Reporting Items for Systematic Reviews and Meta-analyses
UEC: Urgent and emergency care
WOS: Web of Science
