# Supplementary material for: Effects of service changes affecting distance/time to access urgent and emergency care facilities on patient outcomes: a systematic review
Source: BMC Med. 2020 May 20;18:117. doi: 10.1186/s12916-020-01580-3 (PMC7237240; doi:10.1186/s12916-020-01580-3)
Supplement: Supplementary file 1 — Medline search strategy. [file 12916_2020_1580_MOESM1_ESM.docx]

## Appendix 1: MEDLINE search strategy

Database: Ovid MEDLINE(R) and Epub Ahead of Print, In-Process & Other Non-Indexed Citations and Daily <1946 to February 05, 2019>

Search Strategy:

--------------------------------------------------------------------------------

1 *Emergency Service, Hospital/ (37159)

2 *Emergency Medical Services/ (28356)

3 *Emergency Medicine/ (9491)

4 (emergency adj2 service*).ab,ti. (15026)

5 "emergency care".ab,ti. (7936)

6 "urgent care".ab,ti. (1763)

7 "emergency department* ".ab,ti. (79373)

8 "accident and emergency".ab,ti. (4515)

9 casualty.ab,ti. (5181)

10 *Ambulances/ (3459)

11 ambulance$.ab,ti. (9514)

12 "Transportation of Patients"/ (8960)

13 or/1-12 (153712)

14 Rural Health Services/ (11879)

15 (rural$ or island$).ab,ti. (203994)

16 14 or 15 (206752)

17 13 or 16 (356779)

18 ((service$ or health or department$ or deliver$) adj3 (clos$ or chang$ or reorganis$ or merg$ or reconfigur$ or relocat$ or restructur$)).ab,ti. (33564)

19 health facility closure/ or health facility merger/ or health facility moving/ (7163)

20 Health Services Accessibility/ (67829)

21 distance.ab,ti. (207028)

22 access$.ab,ti. (450752)

23 Time Factors/ (1143527)

24 (time$ adj2 travel$).ab,ti. (3348)

25 or/18-24 (1840113)

26 17 and 25 (49092)

27 Myocardial Infarction/ (159731)

28 myocardial infarction.ab,ti. (166461)

29 MI.ab,ti. (42299)

30 heart attack$.ab,ti. (5148)

31 Stroke/ (88836)

32 stroke.ab,ti. (213756)

33 major trauma.ab,ti. (3231)

34 ASTHMA/ (119387)

35 asthma attack$.ab,ti. (2337)

36 (asthma adj3 exacerbation$).ab,ti. (6461)

37 Pulmonary Disease, Chronic Obstructive/ (35064)

38 chronic obstructive pulmonary disease$.ab,ti. (42822)

39 copd.ab,ti. (40387)

40 Pregnancy Complications/ (86598)

41 pregnancy complication$.ab,ti. (5384)

42 Emergency Treatment/ (10211)

43 *EMERGENCIES/ (12339)

44 *Acute Disease/ (8158)

45 or/27-44 (745351)

46 26 and 45 (5124)

47 limit 46 to (english language and yr="2000 -Current") (3960)

***************************

Search step 13 combines the different terms and synonyms for the concept Emergency Care using OR.

Search step 16 combines the different terms for the concept of rural or Island healthcare using OR.

Search step 17 combines the Emergency Care and Rural healthcare terms terms using OR.

Search step 25 combines the different terms for services changes and distance using OR.

Search step 26 combines steps 17 and 25 together using AND.

Search step 45 combines the terms for different emergency medical conditions relevant to the review using OR.

Search step 46 combines steps 26 and 45 together using AND.

Search step 47 limits the search to English Language and the date range 2000-Current.
