# Supplementary material for: Effects of service changes affecting distance/time to access urgent and emergency care facilities on patient outcomes: a systematic review
Source: BMC Med. 2020 May 20;18:117. doi: 10.1186/s12916-020-01580-3 (PMC7237240; doi:10.1186/s12916-020-01580-3)
Supplement: Supplementary file 2 — Data extraction template. [file 12916_2020_1580_MOESM2_ESM.docx]

## Appendix 2: Data extraction template

- Study ID
  - Reference
    *Author year #EN citation (in curly brackets)*
- Country
  - UK
  - Netherlands
  - France
  - Germany
  - Spain
  - Switzerland
  - Other Europe
    *Specify in info box*
  - USA
  - Canada
  - Australia
  - New Zealand
  - Other non-Europe
    *Specify in info box*
  - Multiple countries
  - Unclear/not reported
  - Not applicable
    *Use for systematic reviews*
- Study design
  - Experimental
  - Controlled observational
  - Uncontrolled observational
  - Qualitative
  - Mixed methods
  - Systematic review
  - Other
    *Add brief details in info box*
- Type of setting
  *Where population of interest live*
  - Rural
  - Remote
  - Coastal
  - Mixed
  - Urban/suburban
  - Large city/city region
  - Other
    *Specify in info box*
  - Unclear/not reported
- Population/condition
  - General emergency care
  - Acute MI
  - Stroke
  - Asthma/COPD
  - Major trauma
  - Obstetric/neonatal complications
  - Other
    *Add details in 'info' box*
- Sample source
  *Source from which data were derived, e.g. local audit; national primary care database; hospital records etc.*
  - Data source
- Sample size
  - Number of participants
- Intervention
  - ED closure or relocation
    *Add details in 'info' box*
  - Obstetric unit closure or relocation
  - Centralisation of services
  - Mitigation
    *Intervention aims to mitigate effect of being far away from a UEC facility. Add details in 'info' box*
  - None
    *Study evaluates relationship of distance and outcomes in the absence of reconfiguration*
- Comparator
  - Alternative intervention
  - Earlier time period
  - Baseline
  - Not applicable
- Type of UEC facility
  - Hospital ED
  - Specialist centre
    *E.g. major trauma centre, specialist stroke unit)*
  - Obstetric unit
  - Other
    *Add details in 'info' box*
- Distance/time measure
  - Direct distance
    *Distance 'as the crow flies'*
  - Actual travel distance
  - Travel time
- Type of transport
  - Ambulance/other EMS vehicle
  - Private car
  - Public transport
  - Helicopter
    *Normally excluded*
- Outcomes assessed
  - Mortality
  - Morbidity
    *Add brief details in 'info' box*
  - Hospital admissions
    *Includes avoided admissions/readmissions*
  - Non-conveyance/treatment at scene
  - Qualitative
  - Other
    *Only if main or important outcome*
- Length/period of study
  - Length of study
- Logic model factors
  - Influencing factors
    *Factors affecting time*
  - Explanatory factors
    *Factors affecting patient (and hence health system) outcomes*
- Summary of key results
  - Results
- Key conclusions
  - Conclusions
- Limitations
  - Identified limitations
    *Identified by author or obvious before QA*
