# Supplementary material for: Effects of service changes affecting distance/time to access urgent and emergency care facilities on patient outcomes: a systematic review
Source: BMC Med. 2020 May 20;18:117. doi: 10.1186/s12916-020-01580-3 (PMC7237240; doi:10.1186/s12916-020-01580-3)
Supplement: Supplementary file 3 — Quality assessment results. [file 12916_2020_1580_MOESM3_ESM.docx]

## Appendix 3: Quality appraisal results

| **Study ID** | **Q1** | **Q2** | **Q3** | **Q4** | **Q5** | **Q6** | **Q7** | **Q8** | **Q9** |
| --- | --- | --- | --- | --- | --- | --- | --- | --- | --- |
| *Avdic 2016[6]* | Yes | Yes | Unclear | Yes | Yes | Yes *Registers covered whole population* | Yes | Yes | Yes |
| *Combier 2013[7]* | Yes | Unclear | Unclear | No | No | Yes | Yes | Yes | Yes |
| *El Sayed 2012[8]* | Yes | Yes | Yes | No | Yes | Not applicable *System rather than patient outcomes* | Yes | Yes | Yes |
| *Hansen 2011[9]* | Yes | Yes | Yes | No | No | Not applicable *Health system outcomes* | Yes | Yes | Yes |
| *Hsia 2012[10]* | Yes | Yes | Unclear *Possible changes over time* | Yes | No | Unclear | Yes | Yes | Yes |
| *Hsia 2014[11]* | No *Association between data sets.* | No | No | No *Compares pre and post reconfiguration.* | No | Not applicable *Compares different populations at different time points.* | Yes | Yes | Yes |
| *Knowles 2018[3]* | Yes | No *Heterogeneous population of those attending EDs,* | No | Yes *Control sites* | No | Not applicable | Yes | Yes | Yes |
| *Mustonen 2017[12]* | Yes | Yes | Yes | Yes | No | Not applicable *Health system outcomes* | Yes | Yes | Yes |
| *Roberts 2014[13]* | Yes *In case studies* | Yes | Yes | No | Yes | Not applicable | Yes | Yes | Not applicable *Only descriptive data presented* |
| *Shen 2012[14]* | No *Association using modelling of data sets* | Yes *All AMI patients* | Unclear *All had AMI so may be similar across comparator communities* | No | No | Not applicable | Yes | No *Model estimated effects* | Yes |
| *Shen 2016[15]* | No *Complex associational data, 30 day, 90 day and 1 year mortality.* | Yes *All AMI patients so potentially similar* | Unclear *Potentially as all AMI patients may have been receiving similar care.* | No | No | Not applicable | Yes | Yes | Yes |
| *Yaghoubian 2008[16]* | Yes | Unclear | Yes | No | Yes | Unclear | Yes | Yes | Yes |

Key to questions

1. Is it clear in the study what is the 'cause' and what is the 'effect'? 2. Were the participants included in any comparisons similar? 3. Were the participants included in any comparisons receiving similar treatment/care, other than the exposure or intervention of interest? 4. Was there a control group? 5. Were there multiple measurements of the outcome both pre and post the intervention/exposure? 6. Was follow up complete and if not, were differences between groups in terms of their follow up adequately described and analysed? 7. Were the outcomes of participants included in any comparisons measured in the same way? 8. Were outcomes measured in a reliable way? 9. Was appropriate statistical analysis used?
